# Perioperative Patient Blood Management in Orthopaedics and Traumatology

**DOI:** 10.5704/MOJ.2503.001

**Published:** 2025-03

**Authors:** S Ibrahim

**Affiliations:** Paediatric Orthopaedic Unit, Universiti Kebangsaan Malaysia (UKM) Specialist Children’s Hospital, Kuala Lumpur, Malaysia

In 2021, the World Health Organization issued a policy brief highlighting the urgent need to adopt Patient Blood Management (PBM) globally^1^. PBM is defined as “a patient-centred, systematic, evidence-based approach to improve patient outcomes by managing and preserving a patient’s own blood, while promoting patient safety and empowerment”^2^. However, knowledge of perioperative PBM in Malaysia remains low. In a recent survey of clinicians at a tertiary hospital in Malaysia, 61.5% were found to have inadequate knowledge of PBM^3^.

The three pillars of perioperative PBM focus on: (1) treating pre-operative anaemia, (2) minimising intra-operative blood loss and (3) restricting blood transfusions for post-operative anaemia^4^. Pre-operative anaemia is a significant risk factor, as it increases the likelihood of post-operative complications and mortality due to hypoxia, reduced cardiac perfusion, and impaired wound healing. Additionally, blood transfusions reduce immunity and increases vulnerability to infections^5^.

In this issue of the *Malaysian Orthopaedic Journal*, Sadagatullah *et al* examined the prevalence of blood transfusion and factors influencing blood loss in patients undergoing primary total knee replacement. They found that tourniquet times exceeding two hours significantly increased blood loss and 4.39% of patients required perioperative allogenic blood transfusions due to pre-operative anaemia^6^. A recent study by Schmerler *et al* reported that correcting anaemia pre-operatively reduced allogenic blood transfusion rates from 10.6% in 2010 to 0.6% in 2021 among patients undergoing total knee arthroplasty^7^.

Pre-operative anaemia is common among patients scheduled for elective orthopaedic surgeries. Identifying and treating anaemia prior to surgery can greatly decrease the necessity for transfusions. Effective interventions include oral or intravenous iron supplementation, erythropoiesis-stimulating agents and addressing underlying causes such as chronic kidney disease or nutritional deficiencies^8^.

Surgical techniques to reduce intra-operative and postoperative blood loss, such as the use of tranexamic acid (TXA) have become routine. TXA, an antifibrinolytic agent, significantly decreases blood loss in joint replacements, fracture fixation, and spine surgeries without increasing the risk of thromboembolic events^9^. Hypotensive anaesthesia, haemodilution and cell-savers, reduce bleeding and minimise transfusions in scoliosis surgery^10^.

In the post-operative phase, PBM focuses on optimising oxygen delivery and minimising unnecessary transfusions. Restrictive transfusion thresholds have been associated with better outcomes than liberal transfusion strategies. Mullis *et al* found that a transfusion threshold of 5.5g/dL in asymptomatic patients with musculoskeletal injuries leads to a lower infection rate without an increase in adverse outcomes and no difference in functional outcomes at six months or one year. compared to patients with a transfusion threshold of 7.0g/dL^11^.

Blood transfusions carry considerable risks, including alloimmunization, febrile non-haemolytic reactions, infections, immunosuppression, allergic reactions, transfusion-related acute lung injury (TRALI) and transfusion-associated circulatory overload (TACO)^12^.

Our mentor, the late Professor P. Balasubramaniam, often reminded us, "Treat the patient, not the radiograph." Similarly, we should “treat the patient, not the haemoglobin”. Blood transfusion should not be the primary option to treat perioperative anaemia – oral or intravenous iron are safer alternatives.
